# Host defense triggers rapid adaptive radiation in experimentally evolving parasites

**DOI:** 10.1002/evl3.104

**Published:** 2019-03-05

**Authors:** Sarah E. Bush, Scott M. Villa, Juan C. Altuna, Kevin P. Johnson, Michael D. Shapiro, Dale H. Clayton

**Affiliations:** ^1^ School of Biological Sciences University of Utah Salt Lake City Utah 84112; ^2^ Illinois Natural History Survey, Prairie Research Institute University of Illinois at Urbana‐Champaign Champaign Illinois 61820

**Keywords:** Adaptation, diversification, host switch, camouflage, background‐matching coloration, natural selection, Phthiraptera, rock pigeon, ectoparasite

## Abstract

Adaptive radiation occurs when the members of a single lineage evolve different adaptive forms in response to selection imposed by competitors or predators. Iconic examples include Darwin's finches, Caribbean anoles, and Hawaiian silverswords, all of which live on islands. Although adaptive radiation is thought to be an important generator of biodiversity, most studies concern groups that have already diversified. Here, we take the opposite approach. We experimentally triggered diversification in the descendants of a single population of host‐specific parasites confined to different host “islands.” We show rapid adaptive divergence of experimentally evolving feather lice in response to preening, which is a bird's main defense against ectoparasites. We demonstrate that host defense exerts strong phenotypic selection for crypsis in lice transferred to different colored rock pigeons (*Columba livia*). During four years of experimental evolution (∼60 generations), the lice evolved heritable differences in color. Strikingly, the observed color differences spanned the range of phenotypes found among congeneric lice adapted to other species of birds. To our knowledge, this is the first real‐time demonstration that microevolution is fast enough to simulate millions of years of macroevolutionary change. Our results further indicate that host‐mediated selection triggers rapid divergence in the adaptive radiation of parasites, which are among the most diverse organisms on Earth.

**Impact Summary**

Understanding species diversification is a central theme in biology. Studies of plants and animals from island archipelagos show that different forms evolve in response to divergent natural selection. Such adaptive radiations are also likely to occur among parasites, which live on “host islands.” Here, we take an experimental evolution approach to better understand how adaptive evolution governs diversification from microevolutionary to macroevolutionary scales. We show that anti‐parasite behavior of birds drives rapid and divergent evolution of cryptic coloration in host specific feather lice. Differences we observed in microevolutionary time reflect differences among species of lice parasitizing different species of birds. Our results imply that host defense should be included with competition and predation as major mechanisms driving species divergence.

Adaptive radiation is a major source of organismal diversity (Simpson 1953; Schluter 2000; Nosil and Crespi 2006; Meyer and Kassen 2007; Losos 2010). Ironically, however, the role of this process in parasite diversification remains unclear, despite the fact that parasitism is one of the most common lifestyles on the planet (Price 1980; de Meeus and Renaud 2002; Poulin 2014; Wiens et al. 2015; Jezkova and Wiens 2017). Parasites may adapt and radiate among host species, just as free‐living species adapt, and radiate among islands within archipelagos. Host species are analogous to islands that limit dispersal and gene flow between parasite populations and species. Nevertheless, as in the case of physical islands, the barriers created by host islands are not absolute because even host‐specific parasites occasionally switch host lineages over macroevolutionary time (Ehrlich and Raven 1964; Ziętara and Lumme 2002; Fordyce 2010; Giraud et al. 2010; Johnson et al. 2011; Hardy and Otto 2014; Clayton et al. 2015; Nylin et al. 2017). Host switching can lead to patterns consistent with adaptive radiation in phytophagous insects (Fordyce 2010; Hardy and Otto 2014; Forbes et al. 2017), fungal plant pathogens (Giraud et al. 2010), helminth worms (Ziętara and Lumme 2002), avian brood parasites (Sorenson et al. 2003), and ectoparasitic arthropods (Johnson et al. 2011; Clayton et al. 2015). Host‐mediated selection also appears to drive the adaptive divergence of parasites exposed to varying defensive regimes on different host islands (Ehrlich and Raven 1964; Price 1980; Loker 2012; Wiens et al. 2015). However, this hypothesis has not been tested experimentally because it is difficult to isolate and manipulate components of host defense. We conducted such a test using an unusually tractable host‐parasite system consisting of rock pigeons (*Columba livia*) and their feather lice (Insecta: Phthiraptera: Ischnocera).

Feather lice are host‐specific parasites of birds that feed on the downy regions of feathers, causing energetic stress that leads to a reduction in host fitness through reduced survival and mating success (Clayton et al. 2015). Feather lice depend on feathers for efficient locomotion. Thus, transmission between host individuals usually requires direct contact, such as that between parent birds and their offspring in the nest. However, feather lice can also disperse by hitchhiking phoretically on parasitic flies that are less host‐specific than lice (Harbison and Clayton 2011). As a consequence, lice periodically end up on novel host species (Clayton et al. 2015). Birds combat lice by removing them with their beaks during regular bouts of preening. Lice are thought to escape from preening through background matching crypsis because light colored bird species have light colored lice, whereas dark colored species have dark colored lice (Bush et al. 2010) (Fig. 1A and B). Although these observations suggest that preening is the selective agent responsible for the evolution of cryptic coloration in feather lice, this hypothesis has never been tested experimentally.

**Figure 1:**
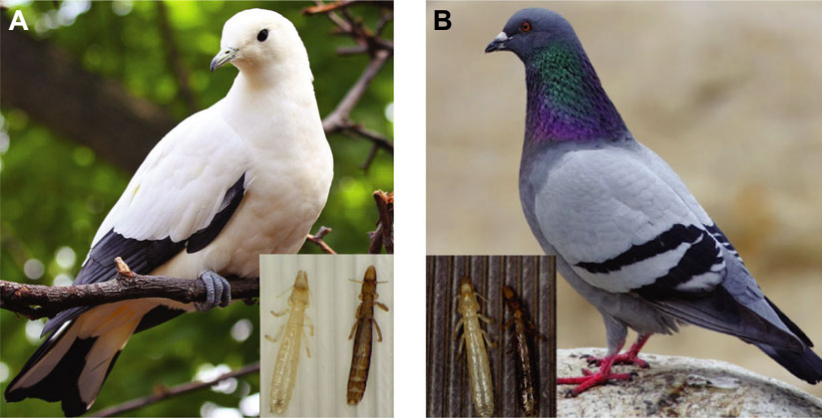
Matching coloration of host‐specific feather lice on pigeons. The light‐colored louse, *Columbicola wolffhuegeli* (left in both insets), parasitizes (A) the Australian pied imperial pigeon, *Ducula bicolor*. The dark‐colored louse, *C. columbae* (right in both insets), parasitizes (B) the cosmopolitan rock pigeon, *Columba livia*. Pigeon photo (A) by Greg Hume (https://commons.wikimedia.org/wiki/File:Pied_Imperial_Pigeon_04.jpg); pigeon photo (B) by Mike Atkinson (reproduced with permission, mikeatkinson.net). Photos of lice by SEB.

## Materials and Methods

### STUDY SYSTEM

One of the challenges of experimental work with host‐specific parasites is that, by definition, they are difficult to culture in sufficient numbers on novel host species. We circumvented this problem by working with rock pigeons (*Columba livia*), a single host species that harbors extensive intraspecific diversity in color as a result of artificial selection (Shapiro and Domyan 2013). Rock pigeons with contrasting plumage colors were used as stepping stones to simulate environments that lice dispersing between different species of pigeons would encounter in nature. All animal procedures were approved by the IACUC of the University of Utah.

### ELIMINATION OF “BACKGROUND” LICE

Before using pigeons in experiments, all “background” lice were eradicated by housing the birds in low humidity conditions (< 25% relative ambient humidity) for ≥ 10 weeks. This method kills lice and their eggs, while avoiding residues from insecticides (Harbison et al. 2008). During experiments, the relative humidity in animal rooms was increased to 35–60%, which provides sufficient humidity for feather lice to extract the moisture they need from the air while living on birds (Nelson and Murray 1971).

### IMPAIRED PREENING

**Figure 2:**
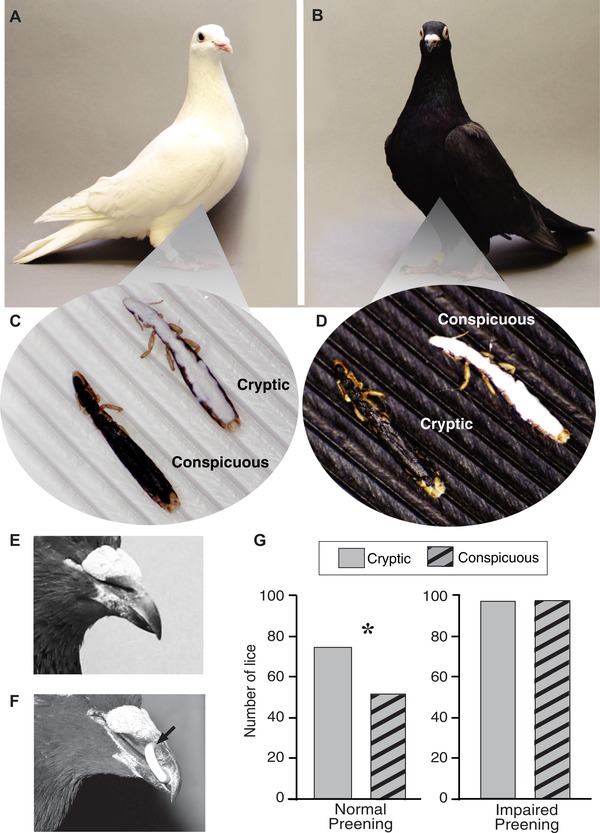
Preening selects for cryptically colored lice. Eight white (A) and eight black (B) rock pigeons were infested with live *C. columbae* that had been painted black or white to make them relatively conspicuous or cryptic, depending on background feather color (C, D). Each of the pigeons, which were isolated in 16 cages, received 30 conspicuous, and 30 cryptic lice, for a total of 960 painted lice across the 16 birds. Half of the white pigeons and half of the black pigeons, chosen at random, could preen normally (E), while the other half were fitted with “poultry bits” to impair preening ability (F, arrow). (G) Pigeons that could preen normally had significantly more cryptic than conspicuous lice at the end of the 48‐h experiment (Fisher's Exact test, *P =* 0.037). In contrast, there was no significant difference in the number of conspicuous and cryptic lice on pigeons with impaired preening (Fisher's Exact test, *P* = 1.0). Photos A&B by Sydney Stringham (reproduced with permission).

Preening was impaired using harmless poultry bits, which are C‐shaped pieces of plastic inserted between the upper and lower mandibles of a bird's beak (Fig. 2F). Bits spring shut in the nostrils to prevent dislodging, but without damaging the tissue. They create a 1–3 mm gap that prevents the forceps‐like action of the bill required for efficient preening (Clayton et al. 2005). Bits have no apparent side effects and they do not impair the ability of birds to feed (Clayton and Tompkins 1995).

### PREENING‐MEDIATED SELECTION EXPERIMENT

*Painting lice*: We manipulated the color of lice by covering the dorsal surface of live, adult *C. columbae* with enamel paint (Floquil^TM^ Railroad Enamel, Vernon Hills, IL). Painting is a reliable method that has been used successfully with other species of lice, even under field conditions (Zohdy et al. 2012). First, we tested whether the paint affects survival of *C. columbae*, as follows: live adult *C. columbae* were removed from infested pigeons and placed in a petri dish next to a small piece of dry ice to keep them anesthetized with CO_2_ during the painting process. We divided 75 lice into three treatments: 25 lice painted white, 25 lice painted black, and 25 unpainted control lice. Paint was applied to the dorsal surface of each louse with a very fine brush. Lice in the control treatment were handled and brushed, but without applying paint. We did not paint the legs of lice, as this might interfere with mobility. Painted lice were placed on feathers from gray feral pigeons in 50 mL glass tubes in a Percival^©^ incubator set at optimal conditions for lice: 33˚C and 75% relative humidity on a 12‐hour light/dark photoperiod (Bush and Clayton 2006). We compared the survival of lice among the three treatments under a dissecting scope (Olympus® SZ‐CTV stereoscope) on six occasions over a 20‐day period (days: 1, 3, 5, 7, 11, and 20). Over this period of time, there was no significant difference in the survival of lice painted white, painted black, or unpainted controls (Kaplan–Meier survival, Wilcoxon *χ* ^2^ = 2.2, df = 2, *P* = 0.34).

*Experimental infestation with painted lice*: 16 pigeons (eight white, eight black; Fig. 2A and B) were randomly divided (within each color treatment) into two preening treatments: half could preen normally, and the other half had their preening impaired with bits (see above). Pigeons in this experiment were housed individually in 30 × 30 × 56 cm wire mesh cages in our animal facility. Cages were separated by plastic partitions to prevent any contact between the feathers of birds in adjacent cages, which might allow transmission of lice between pigeons. Birds were maintained on a 12‐hour light/dark photoperiod and provided ad libitum grain, grit, and water.

Each bird received 30 cryptic lice and 30 conspicuous lice (Fig. 2C and D). The survival of lice was assessed 48 hours after the pigeons were experimentally infested. To do this, all pigeons were sacrificed and their lice removed by “body washing” (Clayton and Drown 2001). Each louse was inspected under a dissecting scope and the number of white and black lice recovered from each bird was recorded.

### EXPERIMENTAL EVOLUTION

To test whether preening selects for divergence in the color of lice, we infested different colored pigeons with normal, unpainted *C. columbae*. Prior to experimental infestation, recipient pigeons were cleared of lice by housing them in low humidity conditions (as described above). Next, we transferred 2400 lice from wild caught feral rock pigeons to 96 captive rock pigeons (25 lice per bird): 32 white pigeons, 32 black pigeons, and 32 “gray” pigeons (controls; see Fig. 1B). Within each color, half the birds, chosen at random, were allowed to preen normally (Fig. 2E), whereas the other half were given bits to impair their preening (Fig. 2F). At this time (Time 0), we also randomly sampled hundreds of lice from the source population on wild caught gray feral pigeons and their luminosity was scored (as described below).

Pigeons were housed in groups of four in 1.8 × 1.5 × 1.0 m aviaries. In summary, the 96 pigeons used in this experiment were housed in 24 aviaries, each containing four birds of the same color and preening treatment (two males and two females per aviary).

During the experiment, all pigeons were maintained on a 12‐hour light/dark photoperiod and provided ad libitum grain, grit, and water. When a bird died during the course of the experiment (a rare occurrence), the lice from the dead bird were transferred to a new parasite‐free pigeon of the same color and sex within 24 hours. *Columbicola columbae* lice can survive for days on a dead bird, but cannot leave the dead bird's feathers under their own power, so few lice were lost.

The experiment ran for four years. Given that *C. columbae* has a mean generation time of 24.4 days (Harbison et al. 2008), this is about 60 generations. Every six months, random samples of lice were removed from pigeons and digitally photographed. Lice were removed by anesthetizing them with CO_2_ (Moyer et al. 2002). After exposure to CO_2_ the feathers of each bird were ruffled over a collection tray. Lice not selected randomly for photography were returned to the bird unharmed. The remaining lice were photographed by placing each louse dorsal side up on a glass slide fitted with a Kodak® Q‐13 white color standard. The lice were immobilized by placing a 22 × 22 mm micro cover slip (VWR®) directly on their body. Digital photographs were taken at high resolution (uncompressed TIFF 2560 × 1920 pixels) using a DP25 digital camera on an Olympus® SZ‐CTV stereoscope linked to a computer running CellSens® image acquisition and analysis software. All of the photos were scored digitally (Villafuerte and Negro 1998; Stevens et al. 2007). For each image, the metathorax was selected and luminosity calculated using the open source imaging software ImageJ 1.3. To correct for slight differences in luminosity due to variation in ambient lighting, we also recorded the luminosity of the color standard immediately adjacent to each louse. We determined how much the photograph of the color standard differed from pure white (luminosity = 255), then added this correction factor to the luminosity score (Bush et al. 2010). Although the digital camera is optimized for human vision, pigeons and humans have similar achromatic spectral sensitivities (Brown and Wald 1964; Bowmaker et al. 1997).

### PLUMAGE COLORATION

We also used digital photography to quantify the plumage luminosity of 23 dead pigeons representing the three colors in the evolution experiment (eight black pigeons, five white pigeons, and 10 gray pigeons). The dorsal and ventral surface of each bird was photographed next to a Kodak® Q‐13 white color standard. High‐resolution (5184 × 3456 pixels) digital photos were taken using a Canon® EOS Rebel® SL1 digital camera. We then highlighted the plumage in each image with the “Quick Selection Tool” in Adobe® Photoshop® CC 2015, and determined the luminosity of the highlighted area. To correct for slight differences in luminosity due to variation in ambient lighting, we also recorded the luminosity of the color standard immediately adjacent to the bird. We determined how much the photograph of the color standard differed from pure white (luminosity = 255), then added this correction factor to the luminosity score for the plumage (Bush et al. 2010). We used the mean plumage luminosity of the dorsal and ventral surfaces of each pigeon in analyses.

### COMMON GARDEN EXPERIMENT

The goal of this experiment was to test for heritability of preening‐mediated changes in the color of lice. We therefore used lice only from the 12 aviaries containing birds with normal preening. Lice of each sex were randomly sampled from each aviary using CO_2_. We marked the lice by clipping setae along the right side of the abdomen and thorax with scissors designed for retinal surgery. Setal clipping is a reliable method that has been used successfully with other species of lice, including under field conditions (Durden 1983). Removal of setae does not influence survival, and the setae do not grow back. After clipping, lice from each aviary were placed on a single gray pigeon with impaired preening. The 12 “common garden” pigeons were isolated in 12 wire mesh cages (30 × 30 × 56 cm). After a period of 48 days, all lice were removed from each of the 12 pigeons using CO_2_ (as described above). At 48 days, most F1 offspring had developed to the adult stage, and could be distinguished from members of the parental cohort, which had clipped setae. In contrast, F2 lice had not yet developed to the adult stage. Thus, using this design, we were able to compare adults of the parental and F1 cohorts of lice on each common garden bird. F1 lice were removed from each bird and digitally photographed and their luminosity scored (as described above). The luminosity of the F1 cohort from each common garden pigeon (*n* = 4–29 lice per bird for a total of 170 lice) was then compared to the luminosity of the parental cohort (*n* = 11–48 lice per aviary, for a total of 303 lice).

## Results and Discussion

We measured the selective effect of preening on the color of pigeon lice (*Columbicola columbae*) by comparing the survival of experimentally manipulated lice placed on different colored rock pigeons (see Materials and Methods). Live lice were painted black or white and distributed evenly among eight black and eight white pigeons (Fig. 2A–D). Half of the birds could preen normally, whereas the other half had their preening ability impaired with harmless bits that prevent complete closure of the beak (Fig. 2E and F). After 48 hours, all birds had their lice removed by body washing (Clayton and Drown 2001). Birds with normal preening had significantly more cryptic lice than conspicuous lice at the end of the experiment (Fig. 2G). Conspicuous lice were 40% more likely be removed by preening, revealing intense selection for cryptic coloration. In contrast, there was no significant difference in the number of cryptic and conspicuous lice on pigeons with impaired preening (Fig. 2G).

This direct demonstration of preening‐mediated selection for crypsis implies that preening leads to the diversification of parasite color among different colored hosts. Because feather lice are permanent parasites that pass their entire life cycle on the body of the host, they can be evolved experimentally under natural conditions on captive birds. Therefore, to test for adaptive divergence in response to host preening, we conducted a four‐year experiment (ca. 60 louse generations) using *C. columbae* isolated on captive rock pigeons of different colors (see Materials and Methods). We transferred lice from wild caught gray feral rock pigeons (Fig. 1B) to white, black, or gray (control) rock pigeons that could either preen normally, or were impaired with bits.

At six‐month intervals, random samples of lice were removed from each pigeon and digitally photographed under identical lighting conditions against a color standard (Villafuerte and Negro 1998). The photographs were used to quantify the luminosity (brightness) of individual lice on a gray‐scale from pixel values of 0 (pure black) to 255 (pure white) (Stevens et al. 2007). We used luminosity—the achromatic component of color—because feather lice vary mainly from light to dark (Bush et al. 2010). We also quantified variation in background coloration by measuring the luminosity of plumage on the white, black, and gray pigeons.

**Figure 3:**
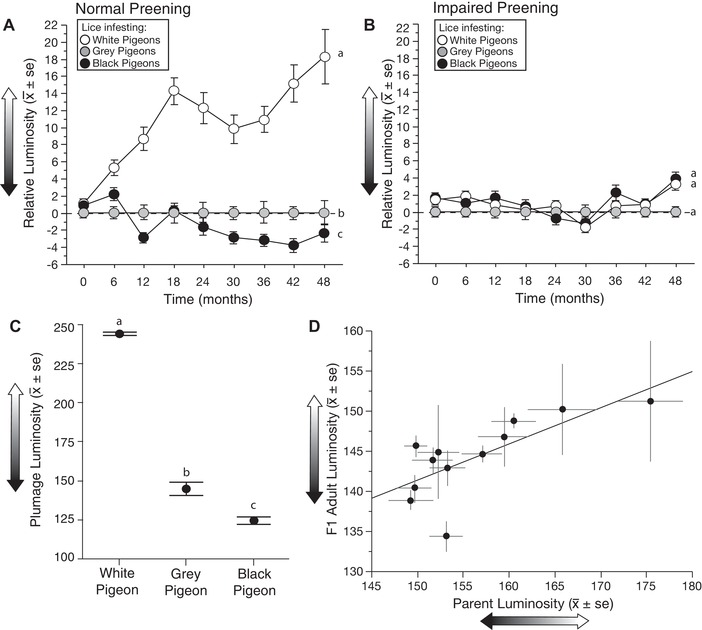
Evolution of feather lice (*C. columbae*) on different colored rock pigeons over a four‐year period (ca. 60 louse generations). (A,B) The *y*‐axis shows changes in the mean (±SE) luminosity (brightness) of lice on white and black rock pigeons, relative to lice on gray rock pigeon controls (set to zero). Different lower case letters indicate statistically significant differences. (A) On birds with normal preening, the relative luminosity of lice on white pigeons increased rapidly (LMM, *P* < 0.0001; Tables A1 and A3); the relative luminosity of lice on black pigeons decreased, but more slowly (LMM, *P* = 0.001; Tables A1 and A3). (B) Relative luminosity did not significantly change over time on white or black pigeons with impaired preening (LMM, *P* ≥ 0.34 in both cases, Tables A2 and A4). (C) Luminosity of plumage from five white, 10 gray (control), and eight black rock pigeons. The three groups differed significantly in luminosity (ANOVA df = 2,22, F = 242.6, *P* < 0.0001; Tukey‐Kramer post hoc tests *P* < 0.001 for all possible comparisons). (D) Common garden experiment showing heritability of preening‐induced changes in the color of lice evolved on different colored pigeons. The adult luminosity of parental and offspring cohorts of lice were highly correlated (linear regression: *r* = 0.72, df = 11, *F* = 11.7, *P* = 0.007).

Over the course of the four‐year experiment, the luminosity of lice on white and black birds changed relative to the luminosity of lice on control gray birds. The relative luminosity of lice on white birds increased dramatically, while the luminosity of lice on black birds decreased, but more slowly (Fig. 3A; Appendix Tables A1 and A3). In contrast, lice on white and black birds with impaired preening showed no significant change in luminosity, relative to lice on control grey birds, even after 60 generations (Fig. 3B; Tables A2 and A4). Thus, merely living and feeding on different colored feathers, in the absence of preening, had no effect on the color of the lice. Changes in the luminosity of lice on preening birds were proportional to differences in background luminosity, that is the luminosity of host plumage. The luminosity difference between gray and white plumage was fivefold greater than that between gray and black plumage (Fig. 3C). Thus, lice on white birds presumably experienced more intense selection for background matching than lice on black birds. Differences in selection intensity may therefore have contributed to the greater change in the color of lice on white birds than on black birds.

Over the course of the experiment, birds with impaired preening had more lice (mean ± SE: 394.5 ± 15.6) than normally preening birds (19.1 ± 2.4) (ANOVA, *F* = 1211.3, *df* = 1, 95, *P* < 0.001), demonstrating that preening is a very effective defense against parasitic feather lice. The mean number of lice per bird varied significantly over time on birds with normal and impaired preening (*P* < 0001, both cases, Table A5). However, the number of lice per bird was not significantly influenced by host color on birds that could preen normally, or on birds that had their preening impaired (*P* ≥ 0.27 in both cases, Table A5).

To further investigate the basis of the observed color changes of lice on birds that could preen normally, we conducted a common garden experiment to test for heritability of the preening‐mediated changes in color (see Materials and Methods). Two years into the four‐year experiment, we removed random samples of lice from white, gray, and black pigeons, marked them by clipping several large hair‐like setae, then transferred the lice to 12 bitted gray pigeons with impaired preening (common garden conditions for lice). Lice remained on the common garden birds for 48 days, which was sufficient time for the marked cohort of lice to breed and for their F1 lice to mature to the adult stage. All adult lice were then removed from each bird and digitally photographed to compare the color of the parental cohort (clipped lice) to adults of the offspring cohort (unclipped lice). Adult luminosity of the parental and offspring cohorts was highly correlated among the 12 common garden birds (Fig. 3D), thus demonstrating that color has a heritable component. This common garden experiment, together with the four‐year experiment (Fig. 3A and B), confirms that the lice on different colored birds evolved changes in luminosity in response to preening‐mediated selection.

**Figure 4:**
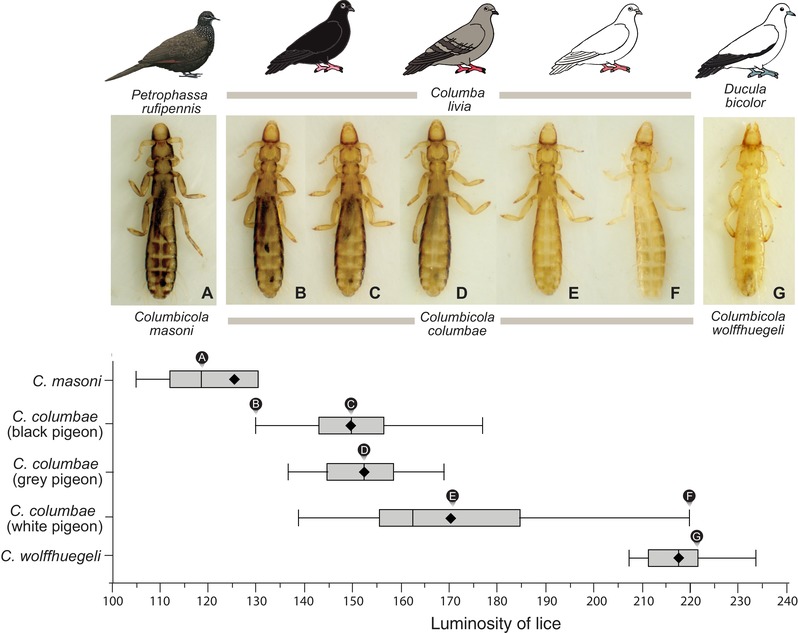
Luminosity of *C. columbae* after four years of experimental evolution on rock pigeons, compared to (A) *C. masoni* from a dark‐colored host species, the Australian chestnut‐quilled pigeon (*Petrophassa rufipennis*), and (G) *C. wolffhuegeli* from a light colored host species, the Australian pied imperial pigeon (also shown in Fig. 1A). Images of experimentally evolved lice in B–F are: (B) one of the darkest *C. columbae* from a black rock pigeon, (C) average *C. columbae* from a black rock pigeon, (D) average *C. columbae* from a gray rock pigeon, (E) average *C. columbae* from a white rock pigeon, and (F) one of the lightest *C. columbae* from a white rock pigeon. Whisker plots show means (diamonds), medians (vertical lines), 1st and 3rd quartiles (boxes), and 1.5 interquartile ranges (whiskers). Letters above whisker plots indicate the luminosity of corresponding photographs. Sample sizes: 7 *C. masoni*, 18 *C. wolffhuegeli*, and 74, 36, and 50 *C. columbae* from black, gray, and white rock pigeons, respectively. Photos of lice by JCA and SMV.

Over the four years of experimental evolution, the increase in mean luminosity of lice on normally preening white pigeons was also accompanied by an increase in variation (Fig. 4). By the end of the experiment, the range in luminosity of lice on white pigeons was much greater than that of lice on gray pigeons (Welch's test of unequal variance *F* = 28.0, *df* = 1, 66.5, *P <* 0.0001). This increase was generated by expansion at the upper end of the scale: 19 of the 50 lice on white pigeons (38.0%) were lighter than any of the lice on gray pigeons. The range in luminosity of experimentally evolved *C. columbae* on white pigeons overlapped the luminosity of *C. wolffhuegeli* (Fig. 4G), the host‐specific parasite of the light‐colored pied imperial pigeon (Fig. 1A). Remarkably, within 60 generations, lice evolved differences in color similar to those of related species that diversified over millions of years (Johnson et al. 2007, Smith et al. 2011). Thus, our study demonstrates rapid adaptive diversification in descendants of a single population living under natural conditions. Our results suggest that the differences in color among wild species of lice may have evolved quickly.

In summary, we show that preening selects for cryptic coloration, and causes the rapid divergence of heritable phenotypes on different host backgrounds. Thus, even small populations harbor sufficient genetic variation for rapid adaptation to novel hosts within several parasite generations. This process is integral to the successful establishment of parasite populations after rare episodes of dispersal to the “wrong” host species, the precursor to host switching. Adaptive radiation catalyzed by host switching is thought to be a central mechanism of diversification among parasites, which represent a substantial fraction of the earth's biodiversity (Price 1980; de Meeus and Renaud 2002; Poulin 2014; Forbes et al. 2017). Our results imply that other modes of host defense, such as immunological resistance, or secondary chemical compounds, may trigger rapid divergence in endoparasites, phytophagous insects, and other hyper‐diverse groups. Host defense should be included with competition and predation as one of the principal mechanisms driving divergence in adaptive radiations.

Associate Editor: A. Charmantier

## Supporting information


**Table A1**. Linear mixed model (LMM) summary comparing the luminosity of lice on pigeons with ***normal preening***.
**Table A2**. Linear mixed model (LMM) summary comparing the luminosity of lice on pigeons with ***impaired preening***.
**Table A3**. Mean luminosity of lice from white, grey, and black pigeons with ***normal preening*** over the course of the four‐year experiment.
**Table A4**. Mean luminosity of lice from black, white, and grey pigeons with ***impaired preening*** over the course the four‐year experiment.
**Table A5**. Repeated‐measures ANOVAs with Huynh‐Feldt Epsilon sphericity correction testing the effect of host color on the number of lice per bird over the 48mo experiment.
