# Supplementary material for: Host defense triggers rapid adaptive radiation in experimentally evolving parasites
Source: Evol Lett. 2019 Mar 5;3(2):120–8. doi: 10.1002/evl3.104 (PMC6457392; doi:10.1002/evl3.104)
Supplement: Supplementary file 1 — Table A1. Linear mixed model (LMM) summary comparing the luminosity of lice on pigeons with normal preening. Table A2. Linear mixed model (LMM) summary comparing the luminosity of lice on pigeons with impaired preening. Table A3. Mean luminosity of lice from white, grey, and black pigeons with normal preening over the course of the four‐year experiment. Table A4. Mean luminosity of lice from black, white, and grey pigeons with impaired preening over the course the four‐year experiment. Table A5. Repeated‐measures ANOVAs with Huynh‐Feldt Epsilon sphericity correction testing the effect of host color on the number of lice per bird over the 48mo experiment. [file EVL3-3-120-s001.docx]

**APPENDIX**

**Table A1**: Linear mixed model (LMM) summary comparing the luminosity of lice on pigeons with ***normal preening***. This LMM is based on the luminosity measurements of 3423 lice sampled over the four-year experiment. Luminosity data at Time 0 are from a random sample of lice drawn from the starting population. Luminosity data for the rest of the experiment (Time 6 mo. - Time 48 mo.) are for lice sampled from 48 individual birds (16 grey control pigeons, 16 white pigeons, and 16 black pigeons) housed in 4 aviaries (4 birds per aviary) for each color treatment. The intercept of the model is set to the value of grey control pigeons at the beginning of the experiment.

| ***Effect of host color on louse luminosity*** | | | | | |
| --- | --- | --- | --- | --- | --- |
| *Random effects* | *Variance* | *Std. Dev.* |  |  |  |
| Individual Bird | 3.182 | 1.784 |  |  |  |
| Aviary | 3.560 | 1.887 |  |  | |
| *Fixed effects* | *Estimate* | *Std. Err.* | *df* | *t value* | *Pr(>\|t\|)* |
| Intercept  Breed (Black Pigeon) | 147.332 | 1.127 | 9.141 | 130.736 | < 0.001* |
|  | -0.253 | 1.584 | 8.926 | -0.160 | 0.877_ |
| Breed (White Pigeon) | 7.480 | 1.586 | 8.970 | 4.715 | 0.001* |
| ***Effect of host color on louse luminosity over time*** | | | | | |
| *Random effects* | *Variance* | *Std. Dev.* |  |  |  |
| Individual Bird | 1.950 | 1.397 |  |  |  |
| Aviary | 1.786 | 1.336 |  |  | |
| *Fixed effects* | *Estimate* | *Std. Err.* | *df* | *t value* | *Pr(>\|t\|)* |
| Intercept | 143.969 | 0.923 | 13.000 | 156.000 | < 0.001* |
| Time | 0.228 | 0.024 | 3354.000 | 9.334 | < 0.001* |
| Time x Breed (Black Pigeon) | -0.107 | 0.033 | 3354.000 | -3.249 | 0.001* |
| Time x Breed (White Pigeon) | 0.286 | 0.033 | 3356.000 | 8.592 | < 0.001* |
| * Indicates significance |  | | |  |  |

**Table A2**: Linear mixed model (LMM) summary comparing the luminosity of lice on pigeons with ***impaired preening***. This LMM is based on the luminosity measurements of 5377 lice sampled over the four-year experiment (Table A4). Luminosity data at Time 0 are from a random sample of lice drawn from the starting population. Luminosity data for the rest of the experiment (Time 6 mo. - Time 48 mo.) are for lice sampled from 48 individual birds (16 grey control pigeons, 16 white pigeons, and 16 black pigeons) housed in 4 aviaries (4 birds per aviary) for each color treatment. The intercept of the model is set to the value of lice on grey control pigeons at the beginning of the experiment.

| ***Effect of host color on louse luminosity*** | | | | | |
| --- | --- | --- | --- | --- | --- |
| *Random effects* | *Variance* | *Std. Dev.* |  |  |  |
| Individual Bird | 0.052 | 0.229 |  |  |  |
| Aviary | 0.533 | 0.730 |  |  | |
| *Fixed effects* | *Estimate* | *Std. Err.* | *df* | *t value* | *Pr(>\|t\|)* |
| Intercept  Breed (Black Pigeon) | 148.542 | 0.435 | 9.055 | 341.623 | < 0.001* |
|  | 1.033 | 0.617 | 9.151 | 1.676 | 0.128_ |
| Breed (White Pigeon) | 0.895 | 0.613 | 8.967 | 1.459 | 0.179_ |
| ***Effect of host color on louse luminosity over time*** | | | | | |
| *Random effects* | *Variance* | *Std. Dev.* |  |  |  |
| Individual Bird | < 0.001 | < 0.001 |  |  | |
| Aviary | 0.602 | 0.776 |  |  | |
| *Fixed effects* | *Estimate* | *Std. Err.* | *df* | *t value* | *Pr(>\|t\|)* |
| Intercept | 144.700 | 0.534 | 19.000 | 270.876 | < 0.001* |
| Time | 0.173 | 0.013 | 5364.000 | 13.046 | < 0.001* |
| Time x Breed (Black Pigeon) | 0.018 | 0.019 | 5363.000 | 0.952 | 0.341_ |
| Time x Breed (White Pigeon) | 0.003 | 0.019 | 5367.000 | 0.173 | 0.863_ |
| * Indicates significance |  | | |  |  |

**Table A3**: Mean luminosity of lice from white, grey, and black pigeons with ***normal preening*** over the course of the four-year experiment. Luminosity data at Time 0 are from a random sample of lice drawn from the starting population. Luminosity data for the rest of the experiment (Time 6 mo. - Time 36 mo.) are for lice sampled from 48 individual birds (16 grey control pigeons, 16 white pigeons, and 16 black pigeons) housed in 4 aviaries (4 birds per aviary) for each color treatment.

|  | **Lice on white pigeons** | |  | **Lice on grey pigeons** | |  | **Lice on black pigeons** | |
| --- | --- | --- | --- | --- | --- | --- | --- | --- |
| **Time (mo)** | *n* | Luminosity ($\bar{x}$ ± se) |  | *n* | Luminosity ($\bar{x}$ ± se) |  | *n* | Luminosity ($\bar{x}$ ± se) |
| 0 | 356 | 143.84 ± 0.50 |  | 342 | 142.76 ± 0.45 |  | 343 | 143.63 ± 0.53 |
| 6 | 132 | 150.29 ± 0.93 |  | 131 | 145.01 ± 0.66 |  | 146 | 147.18 ± 0.76 |
| 12 | 132 | 156.87 ± 1.38 |  | 80 | 148.23 ± 0.87 |  | 148 | 145.30 ± 0.60 |
| 18 | 102 | 165.83 ± 1.53 |  | 79 | 151.56 ± 1.18 |  | 109 | 151.83 ± 0.58 |
| 24 | 127 | 165.67 ± 1.84 |  | 67 | 153.45 ± 1.17 |  | 109 | 151.78 ± 1.00 |
| 30 | 144 | 162.69 ± 1.66 |  | 102 | 152.87 ± 0.75 |  | 106 | 149.98 ± 0.69 |
| 36 | 121 | 160.74 ± 1.54 |  | 51 | 149.85 ± 1.30 |  | 99 | 146.61 ± 0.71 |
| 42 | 71 | 165.39 ± 2.15 |  | 91 | 150.25 ± 0.75 |  | 75 | 146.47 ± 0.78 |
| 48 | 50 | 170.60 ± 3.15 |  | 36 | 152.35 ± 1.39 |  | 74 | 149.98 ± 1.09 |

**Table A4**: Mean luminosity of lice from black, white, and grey pigeons with ***impaired preening*** over the course the four-year experiment. Luminosity data at Time 0 data are from a random sample of lice drawn from the starting population. Luminosity data for the rest of the experiment (Time 6 mo. - Time 36 mo.) are from lice sampled from 48 individual birds (16 grey control pigeons, 16 white pigeons, and 16 black pigeons) housed in 4 aviaries (4 birds per aviary) for each color treatment.

|  | **Lice on white pigeons** | |  | **Lice on grey pigeons** | |  | **Lice on black pigeons** | |
| --- | --- | --- | --- | --- | --- | --- | --- | --- |
| **Time (mo)** | *n* | Luminosity ($\bar{x}$ ± se) |  | *n* | Luminosity ($\bar{x}$ ± se) |  | *n* | Luminosity ($\bar{x}$ ± se) |
| 0 | 348 | 143.86 ± 0.45 |  | 332 | 142.44 ± 0.46 |  | 351 | 144.09 ± 0.56 |
| 6 | 194 | 146.42 ± 0.52 |  | 186 | 144.60 ± 0.55 |  | 185 | 145.61 ± 0.70 |
| 12 | 192 | 149.98 ± 0.63 |  | 186 | 149.18 ± 0.69 |  | 160 | 150.80 ± 0.86 |
| 18 | 186 | 152.12 ± 0.63 |  | 162 | 151.90 ± 0.84 |  | 164 | 152.56 ± 0.82 |
| 24 | 195 | 150.53 ± 0.55 |  | 183 | 149.87 ± 0.64 |  | 161 | 149.31 ± 0.74 |
| 30 | 193 | 151.10 ± 0.51 |  | 166 | 152.92 ± 0.81 |  | 173 | 151.57 ± 0.76 |
| 36 | 195 | 150.27 ± 0.57 |  | 193 | 149.56 ± 0.65 |  | 171 | 151.81 ± 0.92 |
| 42 | 193 | 151.26 ± 0.51 |  | 189 | 150.39 ± 0.59 |  | 184 | 151.17 ± 0.68 |
| 48 | 162 | 154.75 ± 0.65 |  | 195 | 151.53 ± 0.57 |  | 178 | 155.38 ± 0.79 |

**Table A5**: Repeated-measures ANOVAs with Huynh-Feldt Epsilon sphericity correction

testing the effect of host color on the number of lice per bird over the 48mo experiment.

| ***Normally-preening birds*** | | | | | |
| --- | --- | --- | --- | --- | --- |
|  |  |  | *df* | *F- ratio* | *P-value* |
| Color  Time |  |  | 2, 45 | 0.94 | 0.40 |
|  |  |  | 6.1, 272.2 | 16.8 | < 0.0001* |
| Interaction |  |  | 12.1, 272.2 | 1.7 | 0.07_ |
| ***Impaired-preening birds*** | | | | | |
|  |  |  | *df* | *F-ratio* | *P-value* |
| Color |  |  | 2, 45 | 1.36 | 0.27 |
| Time |  |  | 4.8, 217.1 | 64.8 | < 0.0001* |
| Interaction |  |  | 9.6, 217.1 | 1.1 | 0.37 |
| * Indicates significance |  | | |  |  |
